# Supplementary material for: A library-on-library screen reveals the breadth expansion landscape of a broadly neutralizing betacoronavirus antibody
Source: bioRxiv. 2024 Jun 10:2024.06.06.597810. Preprint. [Version 1] doi: 10.1101/2024.06.06.597810 (PMC11195093; doi:10.1101/2024.06.06.597810)
Supplement: Supplement 1 [file media-1.pdf]

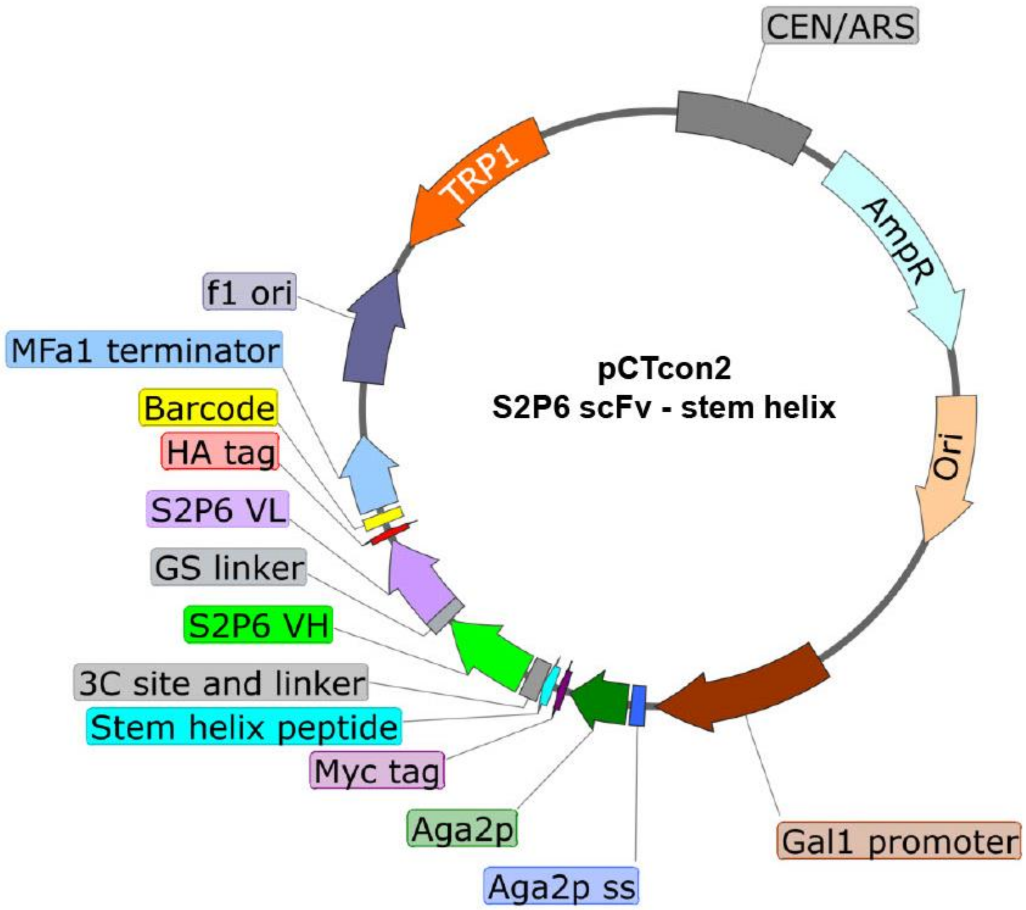

Figure S1. Plasmid map details. Schematic of our plasmid construct for yeast display library-on-library screen with key features highlighted.

A

```
                                           1                              32               48      55 56
UCA_VH   QVQLVQSGAEVKKPGASVKVSCKASGYTFTSYYMHWVRQAPGQGLEWMGIINPSGGSTSY
S2P6_VH  EVQLVQSGAEVKKPGASVKVSCKASGYTFTSQYMHWVRQAPGQGLEWIGIINPSGVHTSY

                  69       78
UCA_VH   AQKFQGRVTMTRDTSTSTVYMELSSLRSEDTAVYYCARGSPKGAFDYWGQGTLVTVSS
S2P6_VH  AQKFQGRVTLTRDTSTSTLYMELSSLRSEDTAVYYCARGSPKGAFDYWGQGTLVTVSS
```

B

```
            4 5                       29 31
UCA_VL   EIVLTQSPGTLSLSPGERATLSCRASQSVSSSYLAWYQQKPGQAPRLLIYGASSRATGIP
S2P6_VL  EIVMMQSPGTLSLSPGERATLSCRASQSVRSNYLAWYQQKPGQAPRLLIYGASSRATGIP

                                                      105
UCA_VL   DRFSGSGSGTDFTLTISRLEPEDFAVYYCQQYGSSPPRFTFGPGTKVDIK
S2P6_VL  DRFSGSGSGTDFTLTISRLEPEDFAVYYCQQYGSSPPRFTFGPGTKVEIK
```

C

scFv residue	Variants in library
V_H 32	Y, Q
V_H 48	M, I
V_H 55	G, V
V_H 56	H, S
V_H 69	M, L
V_H 78	V, L
V_L 4	L, M
V_L 5	T, M
V_L 29	S, R
V_L 31	S, N

Figure S2. S2P6 variant library design. (A-B) Sequence alignments of **(A)** S2P6 V_H and UCA, as well as **(B)** S2P6 V_L and UCA. Somatic hypermutations are highlighted in red. **(C)** Amino acid variants in the S2P6 variant library.

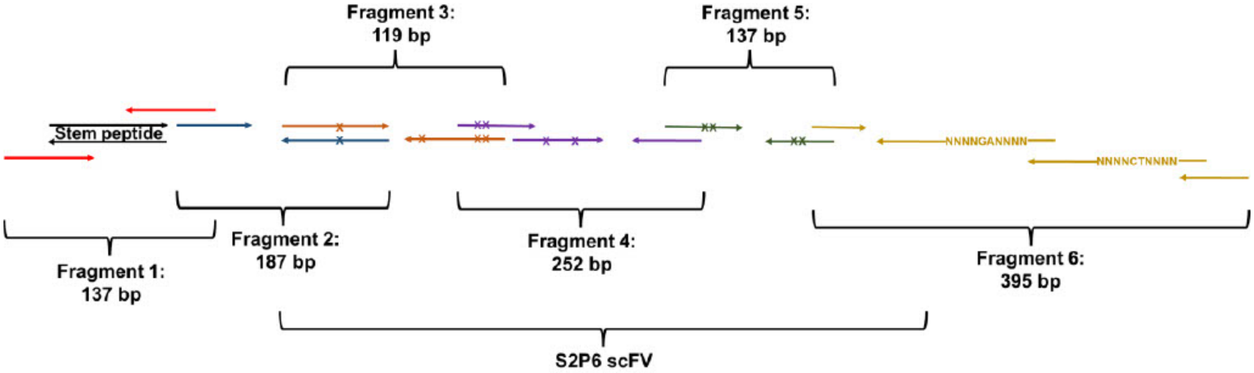

Figure S3. Strategy for assembling the combination variant library. The combination variant library was assembled via overlap PCR of six fragments (represented by the color of primers) encoding (from 5' to 3') the stem helix peptide variant library, S2P6 variant library, linkers, and 16-nucleotide barcode (NNNNGANNNN … NNNNCTNNNN).

A

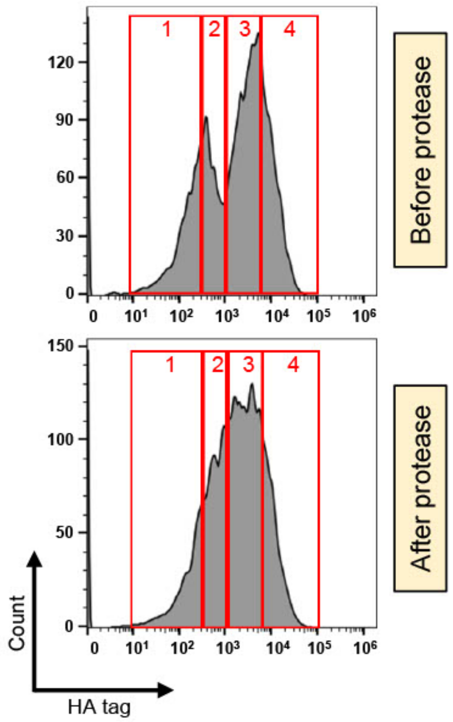

B

Protease	Replicate	Gate	Cell count
Before	1	1	319,217
Before	1	2	317,819
Before	1	3	554,411
Before	1	4	403,008
Before	2	1	318,096
Before	2	2	324,247
Before	2	3	573,971
Before	2	4	472,848
After	1	1	351,057
After	1	2	847,861
After	1	3	1,453,835
After	1	4	563,505
After	2	1	321,741
After	2	2	795,598
After	2	3	1,694,468
After	2	4	845,198

Figure S4. Cell sorting of the combination variant library. (A) Gating scheme for cell sorting of the combination variant library. Four gates were used (i.e. 1, 2, 3, 4) where 1 represents lowest HA tag signal (Alexa Flour 647 nm) and 4 represents highest HA tag signal. Four library samples were sorted in total: two before protease cleavage and two after protease cleavage. **(B)** Cell counts listed for each sample.

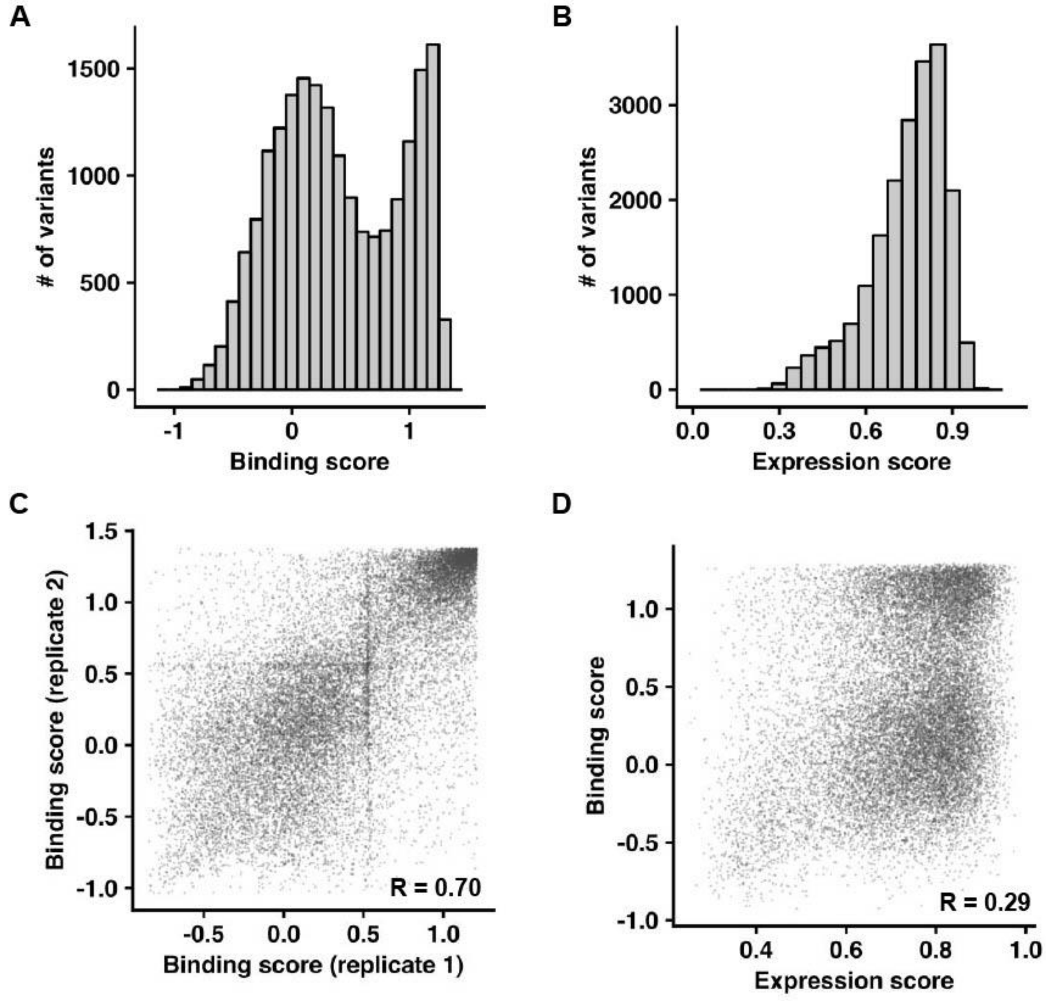

Figure S5. Expression and binding scores of individual variants in the combination variant library. (A) Binding score distribution. **(B)** Expression score distribution. **(C)** Comparison of binding scores between two experimental replicates. **(D)** The relationship between binding score and expression score.

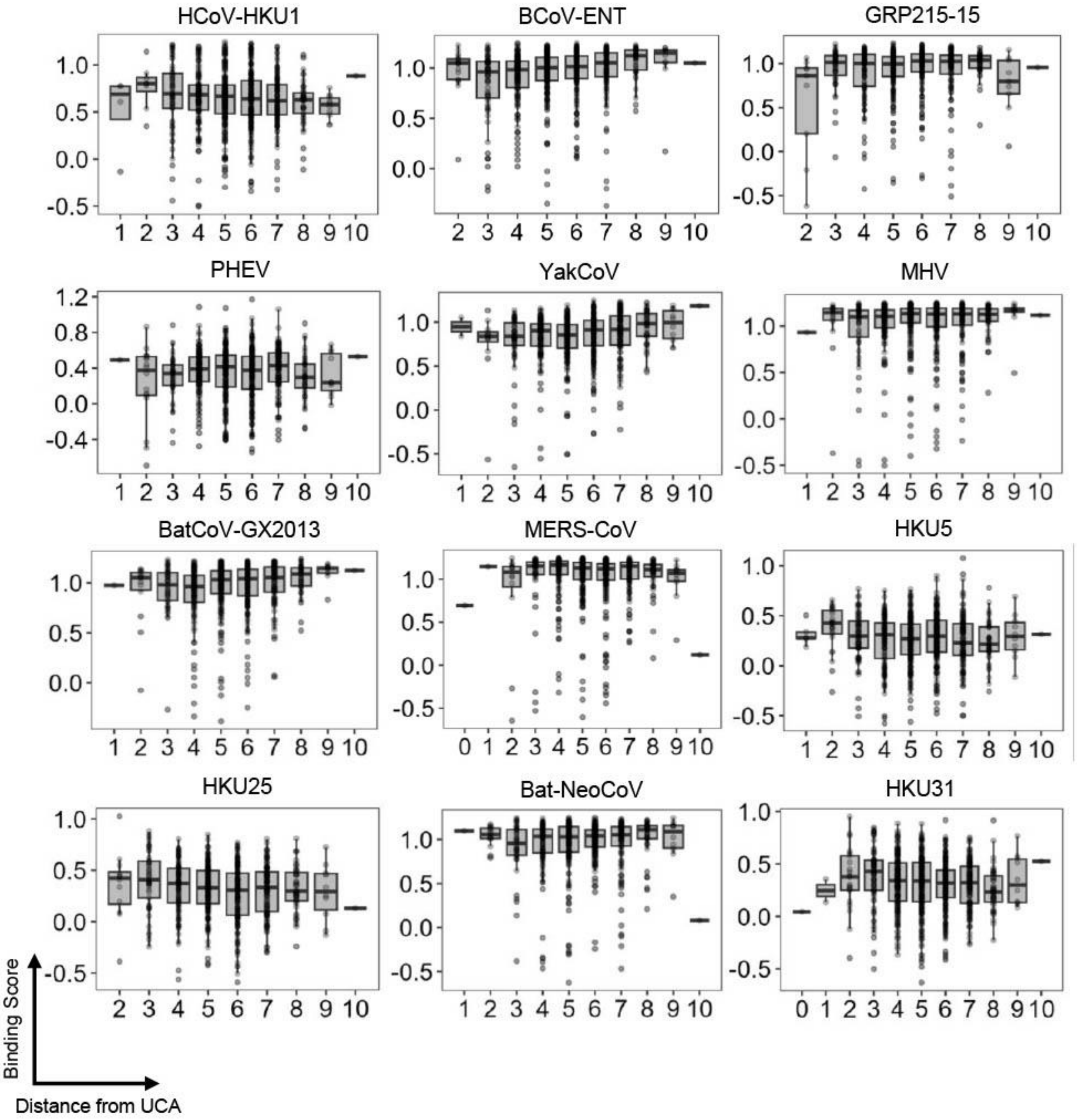
HCoV-HKU1
BCoV-ENT
GRP215-15
PHEV
YakCoV
MHV
BatCoV-GX2013
MERS-CoV
HKU5
HKU25
Bat-NeoCoV
HKU31
Binding Score
Distance from UCA

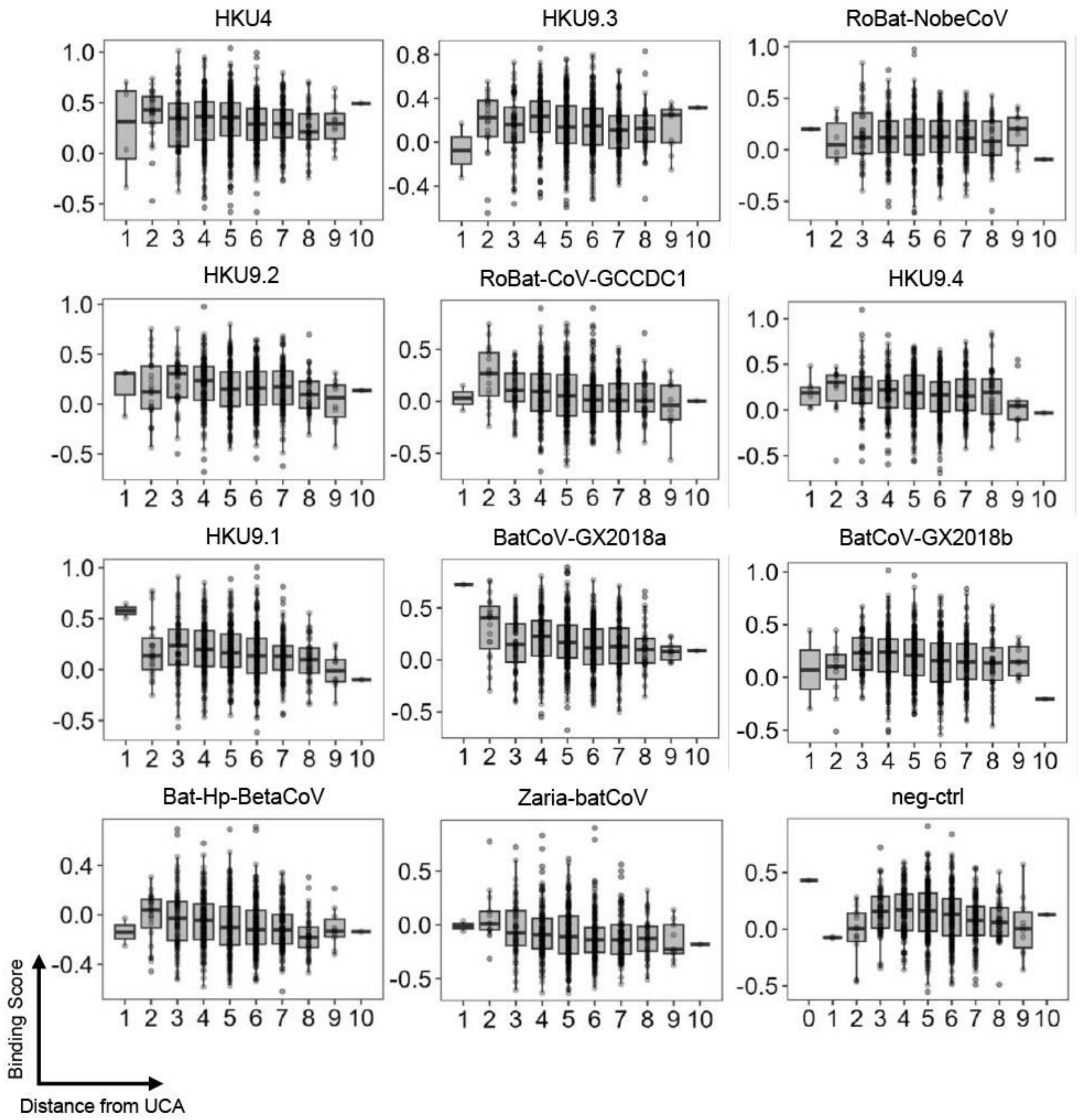

Figure S6. Relationship between binding score and distance from UCA. Binding scores of S2P6 variants with different numbers of somatic hypermutations (i.e. distance from UCA) to the indicated stem helix peptides are shown. Each data point represents the one S2P6 variant. For the boxplot, the middle horizontal line represents the median. The lower and upper hinges represent the first and third quartiles, respectively. The upper whisker extends to the highest data point within a 1.5× inter-quartile range (IQR) of the third quartile, whereas the lower whisker extends to the lowest data point within a 1.5× IQR of the first quartile.

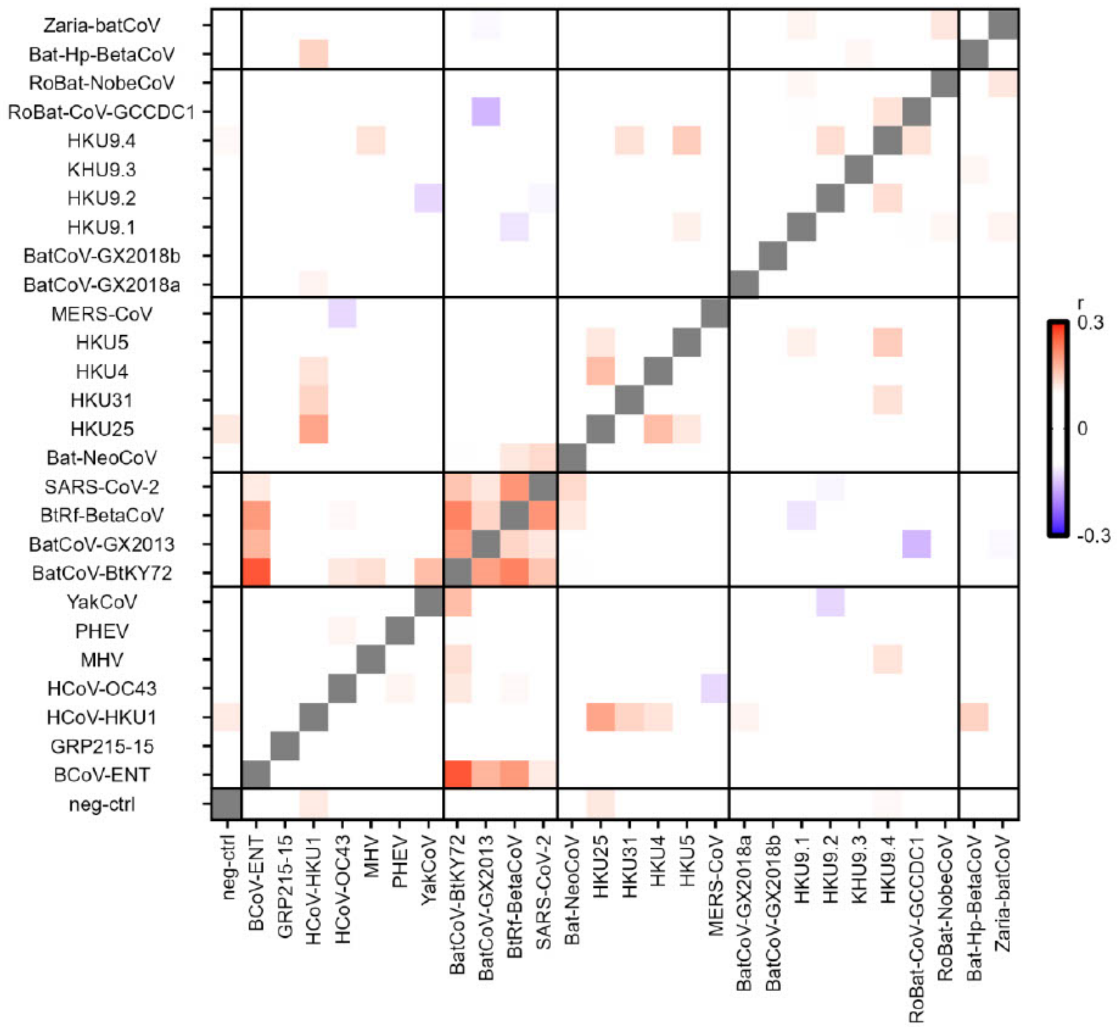

Figure S7. Correlation of binding scores between stem helix peptides. Pairwise Pearson correlations of binding scores between stem helix peptides. Red indicates positive correlation coefficient, whereas blue indicates negative correlation coefficient.

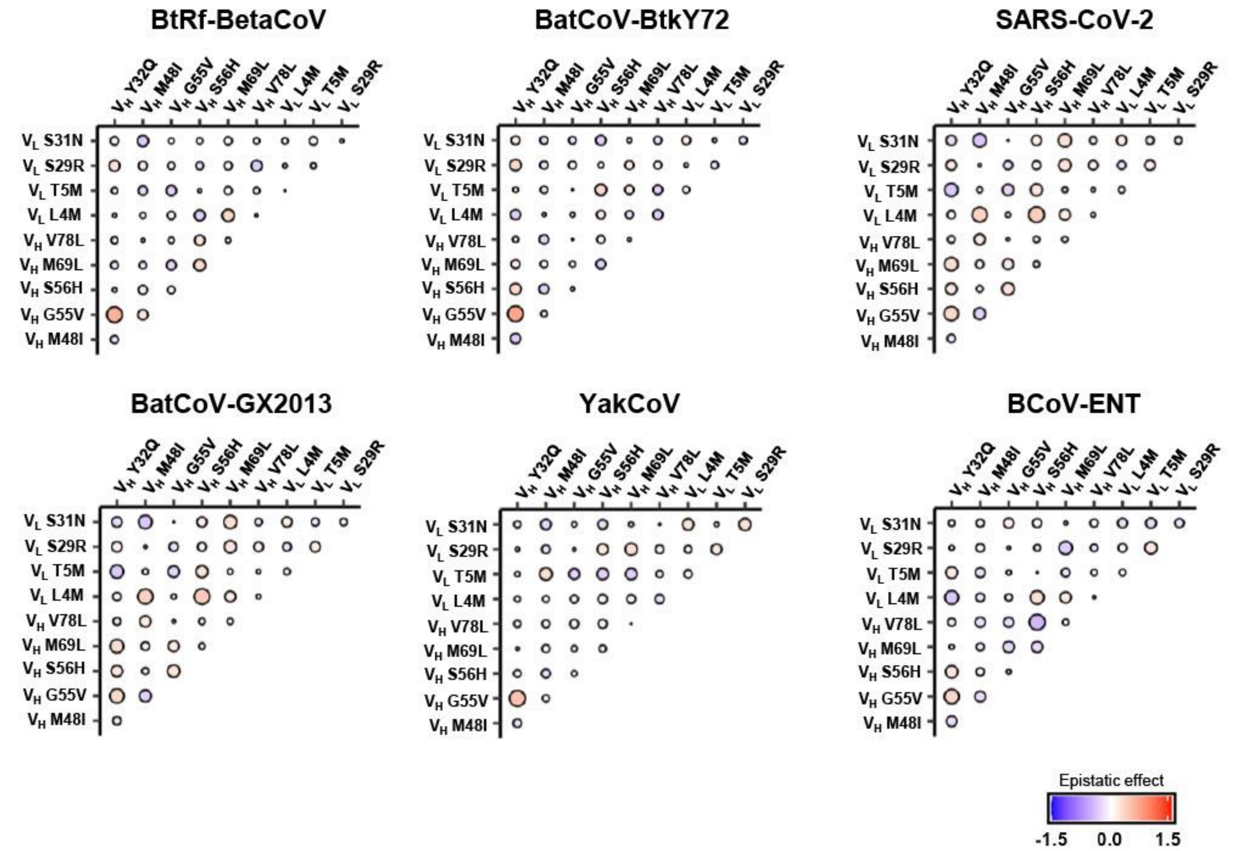

Figure S8. Pairwise epistasis between S2P6 somatic hypermutations. Inference of epistatic interactions between S2P6 somatic hypermutations for binding to BtRf-BetaCoV, BatCoV-BtkY72, SARS-CoV-2, BatCoV-GX2013, YakCoV, and BCoV-ENT. Red and blue represents positive and negative epistasis, respectively. Magnitude is proportional to the size of the circle.

BatCoV-BtkY72

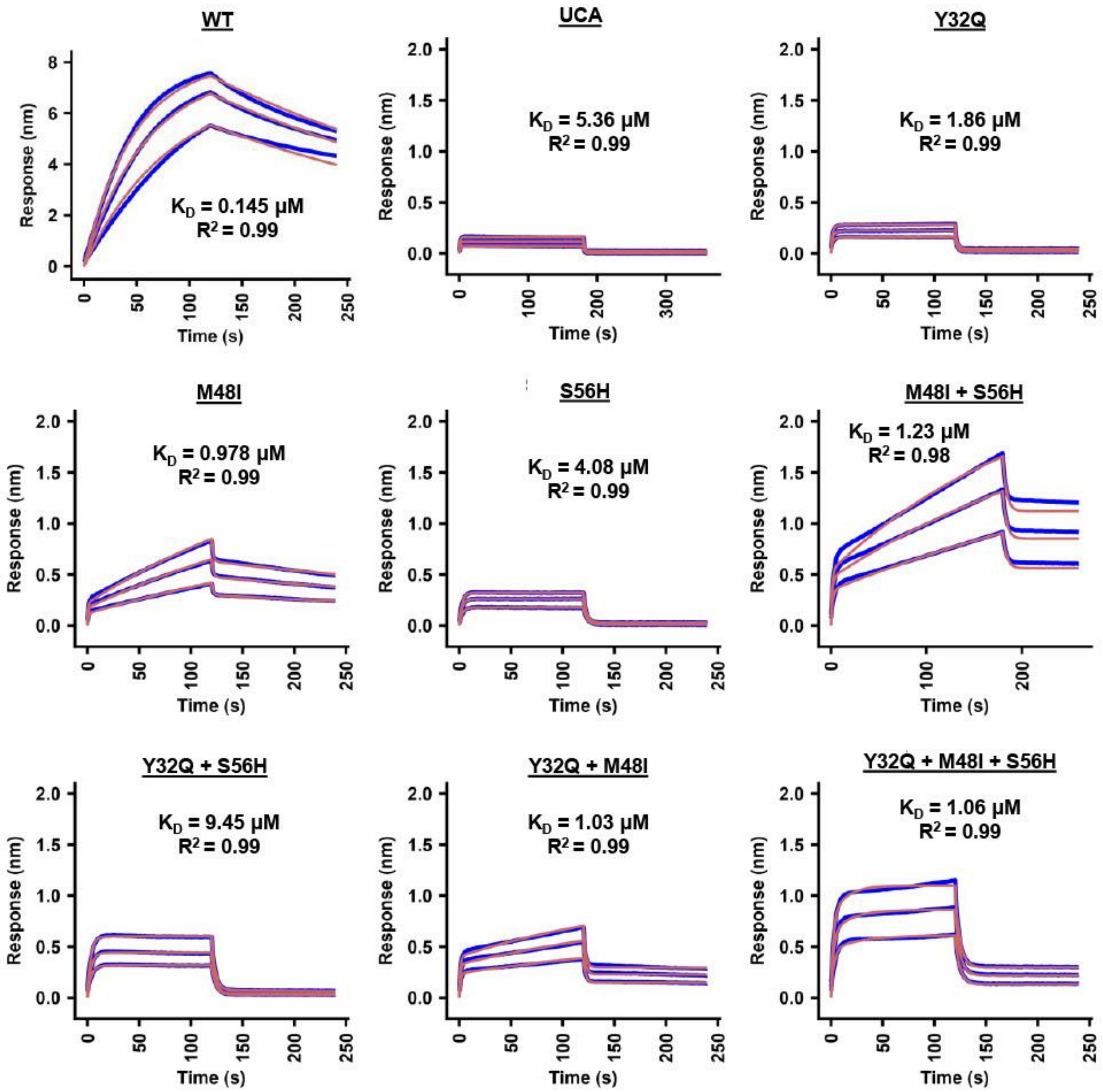

SARS-CoV-2

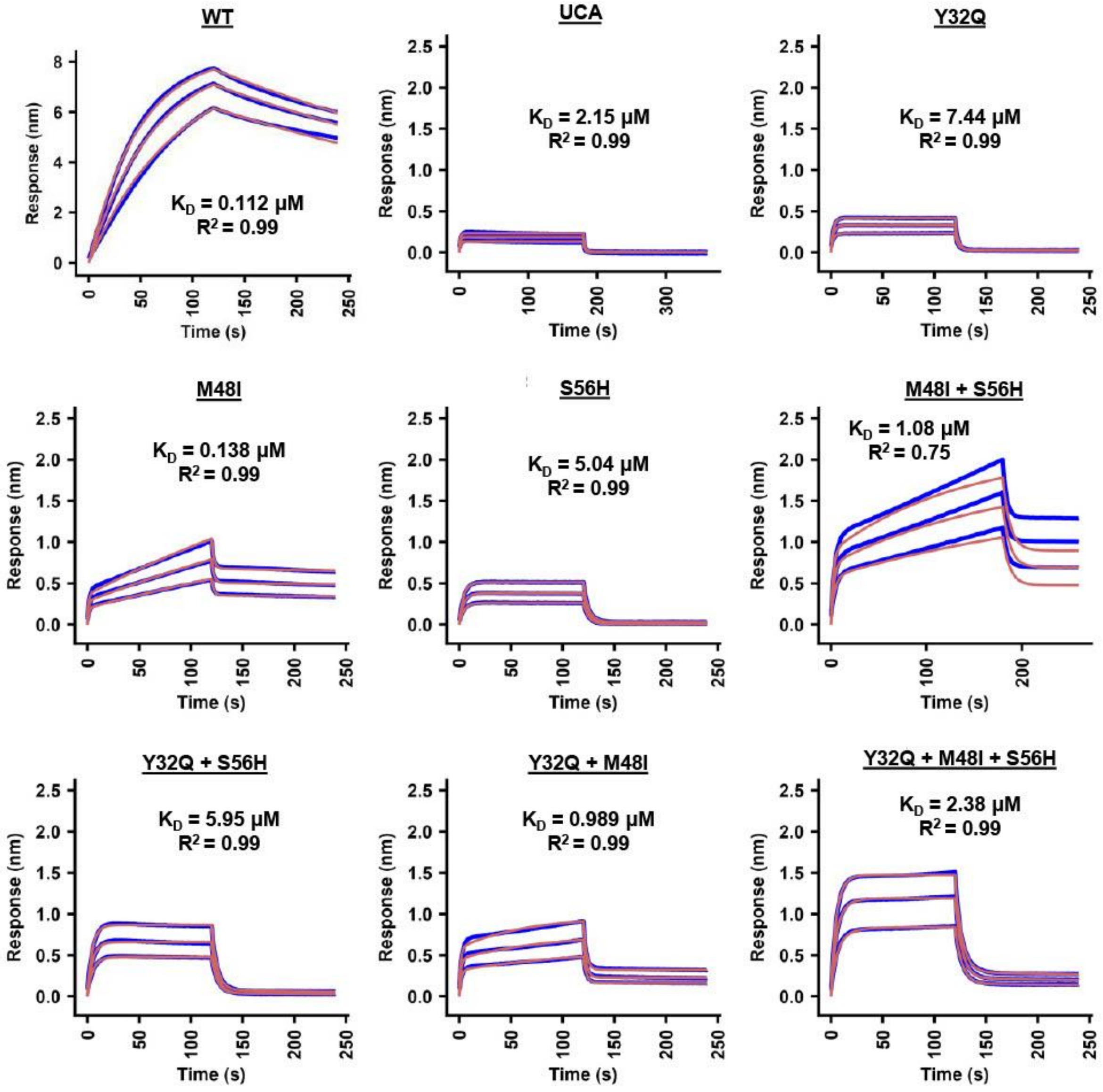

WT
K_D = 0.112 µM
R^2 = 0.99
UCA
K_D = 2.15 µM
R^2 = 0.99
Y32Q
K_D = 7.44 µM
R^2 = 0.99
M48I
K_D = 0.138 µM
R^2 = 0.99
S56H
K_D = 5.04 µM
R^2 = 0.99
M48I + S56H
K_D = 1.08 µM
R^2 = 0.75
Y32Q + S56H
K_D = 5.95 µM
R^2 = 0.99
Y32Q + M48I
K_D = 0.989 µM
R^2 = 0.99
Y32Q + M48I + S56H
K_D = 2.38 µM
R^2 = 0.99
Response (nm)
Time (s)

BtRf-BetaCoV

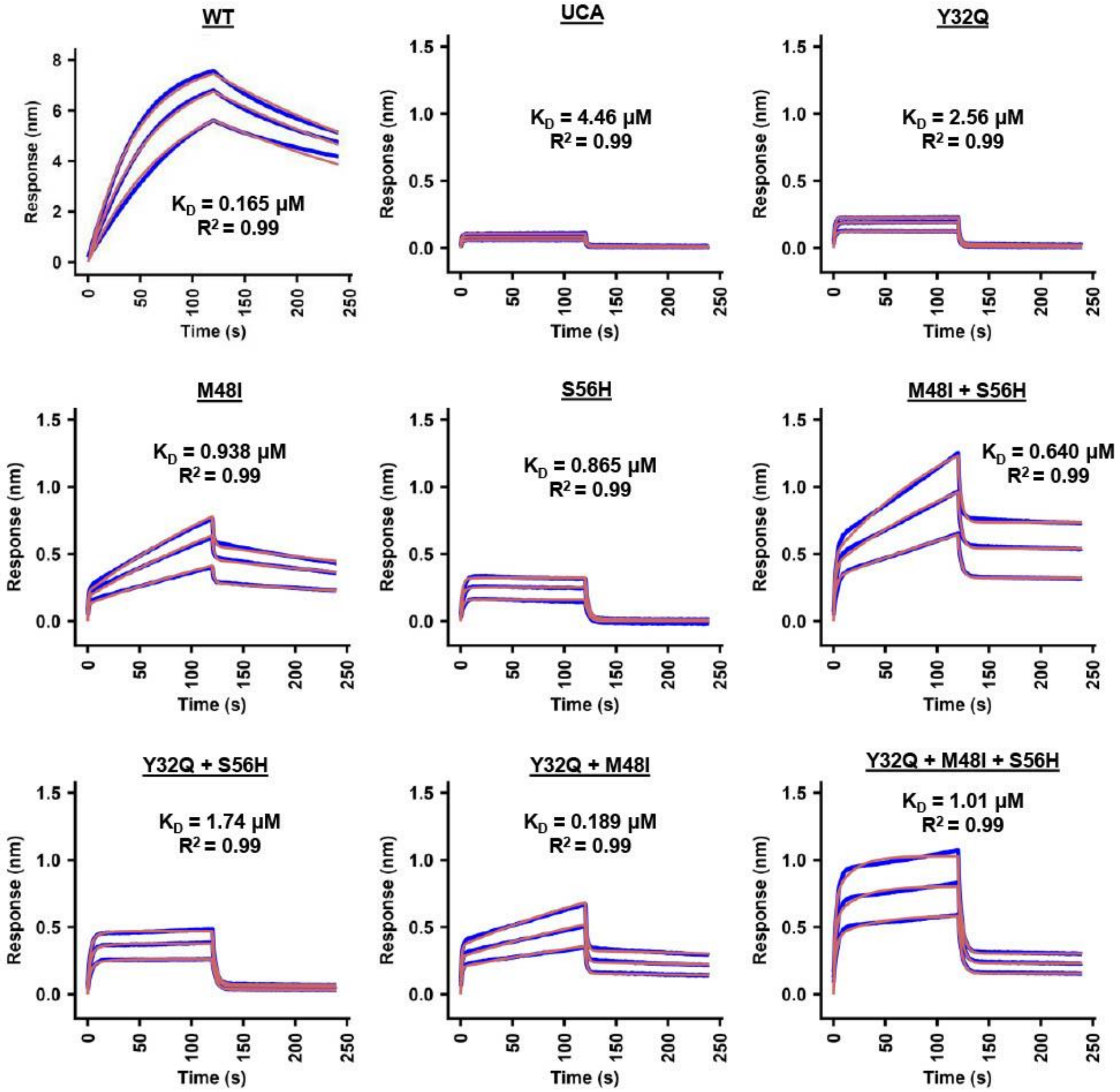

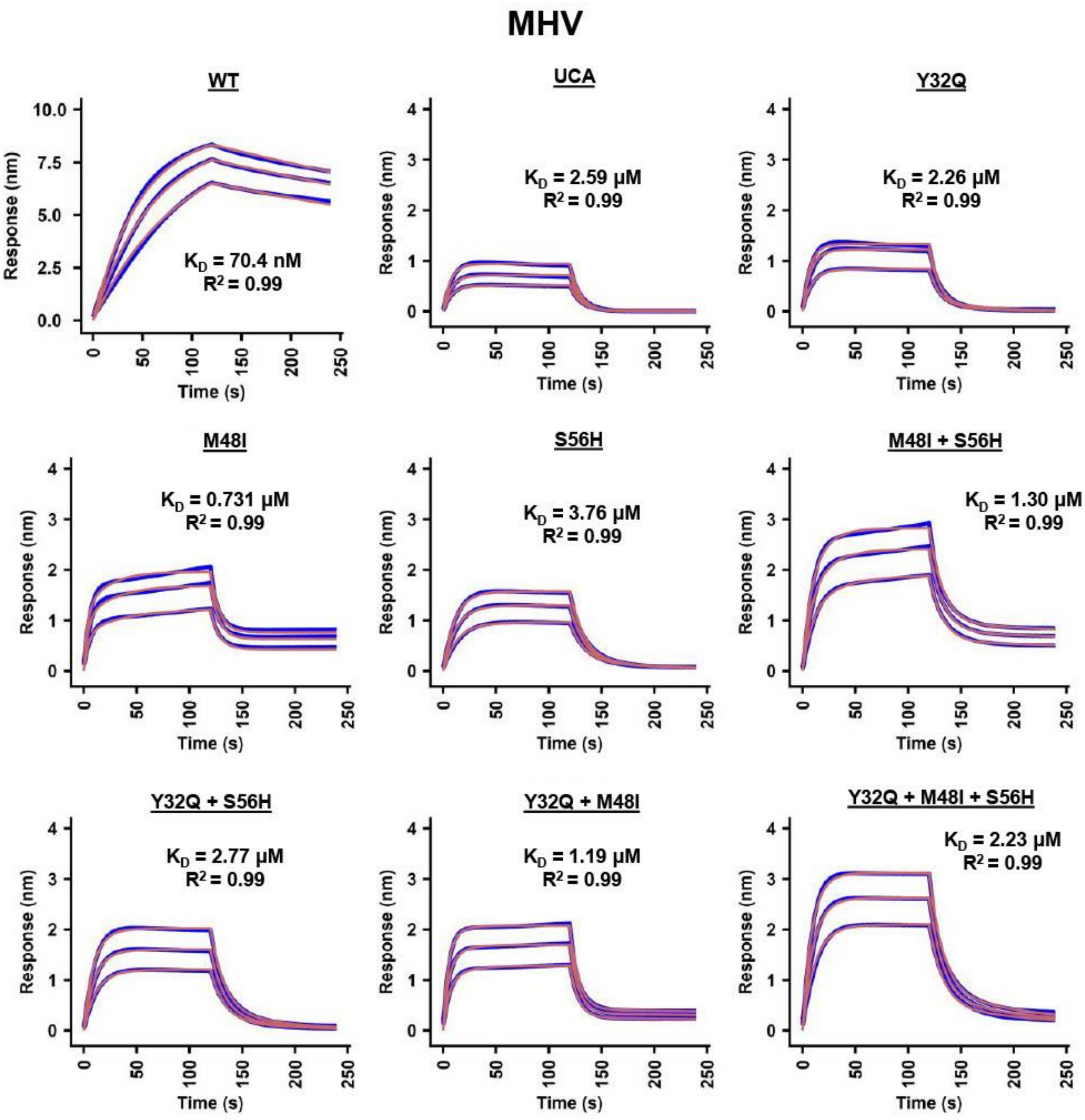

Figure S9. Binding kinetics of recombinantly expressed S2P6 variants. Biolayer interferometry sensorgrams of Fabs (S2P6 WT, UCA, and UCA mutants) binding to BatCoV-BtkY72, SARS-CoV-2, BtRf-BetaCoV, MHV, and HCoV-HKU1 stem helix peptides. Blue lines represent response curve and red lines represent best fit model (1:1 binding model or 2:1 heterogeneous ligand model). Binding kinetics were measured for three Fab concentrations (500 nM, 750 nM, and 1000 nM). Dissociation constant (K_D) and the goodness of model fitting (R^2) are included on each plot.
